# Thermal expansion behavior of thin films expanding freely on water surface

**DOI:** 10.1038/s41598-019-43592-x

**Published:** 2019-05-08

**Authors:** Jae-Han Kim, Kyung-Lim Jang, Kwangho Ahn, Taeshik Yoon, Tae-Ik Lee, Taek-Soo Kim

**Affiliations:** 1 Department of Mechanical Engineering, KAIST, Daejeon, 34141 Korea; 2 Korea Atomic Energy Research Institute, Daejeon, 34057 Korea

**Keywords:** Characterization and analytical techniques, Mechanical engineering, Characterization and analytical techniques

## Abstract

Coefficient of thermal expansion (CTE) for thin film has been measured only from change in thickness because thin film has to be constrained on a solid substrate. However, thin film CTE shows different values depending on the supporting solid substrate. Here, a novel measurement method is suggested to quantitatively measure the in-plane thermal expansion of thin films floating on a water surface. In-plane thermal expansion of thin films on water surface is achieved by heating the water. The CTE is measured through a digital image correlation (DIC) technique. The DIC tracks displacement marks deposited on the film surface, and the in-plane thermal strain is defined as the change in distance between the patterns. The method can be applied to measure the CTE of polymer, metal, and graphene with a thickness ranging from a micrometer to one-atom-thickness. The in-plane thermal expansion of the polystyrene (PS) thin film decreased as the film thickness decreased. The negative CTE of graphene is also successfully explored without any substrate effects or complicated calculations. The CTE measurement method can provide understanding of the intrinsic thermal expansion behavior of thin films including emerging two-dimensional materials.

## Introduction

Thin films may have physical properties different from those of bulk material. One of the physical properties that shows a thickness dependence is CTE^1,2^. CTE is a fundamental material property that represents a change in length of a material per unit temperature change. In thin film systems, a CTE mismatch between adjacent layers in systems can lead to structural deterioration such as interfacial delamination or cracking. Therefore, the intrinsic CTE of thin film should be quantitatively investigated to design robust thin film systems. In conventional CTE measurement methods, however, the thin film CTE has only been measured when the thin film is either fully supported or partially constrained on the solid substrate^1–7^. The substrate constraint can lead to additional calculations to obtain a thin film CTE and to poor reproducibility of CTE data^1^. Above all, the thin film is laterally constrained and expands along the thickness direction only due to the solid substrate. To measure the out-of-plane thermal strain, indirect characterization methods such as spectroscopic ellipsometry^1,5–7^, X-ray reflectometry^2,8^, and neutron reflectometry^9^ have been adopted. Lateral constraint of thin films affects the thermal expansion in the thickness direction. Therefore, Poisson’s ratio effect should be considered to obtain the intrinsic CTE of the thin film^1,5,10^. Moreover, due to interactions between the thin film and the solid substrate, inconsistent magnitude and tendency of thermal expansion have been observed depending on the type of substrate^1,2,5,8,10^. To minimize the interaction, the thin film has been suspended on a solid substrate with a hole^1,6,7^ or a trench^3^. Although the thin film is in free-standing form over the opening, the solid substrate also expands when the temperature changes. Therefore, during the CTE measurement, the thermal expansion of the solid substrate must be deducted from the measured total thermal expansion. Also, poor reproducibility has been shown because of a CTE mismatch between the thin film and the solid substrate^1^.

**Figure 1:**
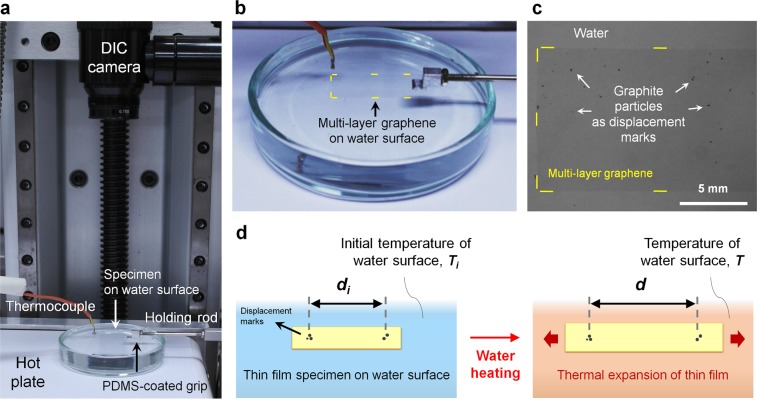
Experimental set-up of in-plane CTE measurement. (**a**) The CTE measurement system consists of a DIC system, a thermocouple, a hot plate, and a holding rod with PDMS-coated grip on an anti-vibration table. A glass petri dish with a thin film specimen is positioned on a hot plate. The thin film is fixed by the van der Waals adhesion of PDMS coating at the end of a holding rod. Thermal expansion is observed through a DIC camera and temperature is measured by a thermometer during the heating. (**b**,**c**) Multi-layer graphene floating on water surface. The yellow lines show the boundary of the multi-layer graphene. Fine graphite particles (tiny black spots) are patterned on the multi-layer graphene as displacement marks in (**c**). (**d**) Schematic illustration of the thermal expansion of the thin film specimen. The thermal strain is defined by a change in the distance between the displacement marks. CTE is expressed in *α* = (*d* − *d*i)/*d*i(*T* − *T*i).

In this work, we present measurements of in-plane thermal strain on a water surface without any substrate constraints (Fig. 1a,b). The CTE measurement system for the thin films on the water surface is shown in Fig. 1a. The water enables frictionless sliding of the thin films on the surface^11^, thereby enabling the thin film to expand freely on the water surface in the lateral direction without any substrate constraints^12^. The water is also used as a heating platform for the thin films by increasing the water temperature using a hot plate. Accordingly, the in-plane thermal expansion of thin films floating on the water surface is simply achieved by heating the water. The DIC technique has been utilized for direct *in-situ* measurements of the in-plane thermal expansion of thin films. Through the DIC technique, displacement marks deposited on the film surface (Fig. 1c) were tracked and changes in distance between the marks were calculated as thermal strain (Fig. 1d). The CTE of the thin films was determined according to the in-plane thermal strain with respect to the temperature change. Our method provides direct and rapid CTE measurement, whereas conventional methods require an additional calculation step to eliminate the substrate effect^1–7^ or data analysis to determine changes in the film thickness^1,13,14^. To validate our methodology, we perform CTE measurements on poly(dimethylsiloxane) (PDMS), PS, poly(methyl methacrylate) (PMMA), and Ag films for polymer and metal thin films. The effect of chain conformation on the in-plane thermal expansion behavior of PS thin film was experimentally explored. Furthermore, in-plane CTE of graphene was also successfully investigated. Our method can be used to explore the in-plane thermal behavior of thin films without any constraints from adjacent solid substrates, thereby enabling the thin film to expand freely on water surface.

## Results

### Fabrication of thin films on water surface

A thin film specimen on a water surface was prepared through fabrication, pattern deposition, peel-off, etching and transfer processes (Supplementary Fig. S1). Thin film was fabricated on a Cu/SiO_2_/Si substrate and the details of how each specimen was fabricated are provided in the Methods section. To measure the thermal strain through the DIC camera, displacement marks were patterned on the thin film using fine graphite particles. After the pattern deposition, the film/Cu layer was delaminated from the SiO_2_/Si substrate using a water-assisted peel-off. The Cu sacrificial layer was etched by a Cu etchant solution, which was a 0.05 M ammonium persulfate solution. Then, the thin film specimen scooped onto the deionized water surface. After preparing the thin film specimen, a glass petri dish with floating thin film was placed on a hot plate (Fig. 1a,b). The edge of the thin film specimen was held by the PDMS-coated grip. The van der Waals adhesion between the thin film and the PDMS enables easy manipulation of the thin films on the water surface without any slippage or damages^11^. The water was heated by a hot plate until the temperature reached about 350 K. The temperature of the water surface was recorded through a thermocouple that was connected to a thermometer. As for possible variation between the water surface temperature and the film temperature, finite element analysis was conducted. The results (Supplementary Fig. S2) showed that the thin film immediately heated up to the temperature of the water surface. Therefore, due to the thin film’s small thickness, its temperature can be considered to be identical to the temperature of the water. Measurement of temperature was conducted near the specimen at the center of the dish. It was found that the position dependence of the temperature was negligible if the thermocouple was positioned near the center of the water (Supplementary Fig. S3). During the heating process, the thermal strain of the thin film was measured in real time through a DIC device. The DIC technique provides accurate measurement of thermal strain by pattern matching and tracking^15,16^. The thermal strain was calculated by dividing the difference between pattern distance *d* at temperature *T* and initial pattern distance *d*_*i*_ at initial temperature *T*_*i*_ by the *d*_*i*_. (Fig. 1c,d). With a temperature above 350 K, the thermal strain was hard to measure because the temperature was approaching the boiling temperature of water. Near the boiling temperature, small bubbles started to form in the water, and soon wavelets were generated due to the popping of bubbles. This limitation can be overcome by replacing the type of liquid and future studies will utilize various liquid platforms that have boiling temperatures higher than that of water.

### Validation of in-plane CTE measurement

**Figure 2:**
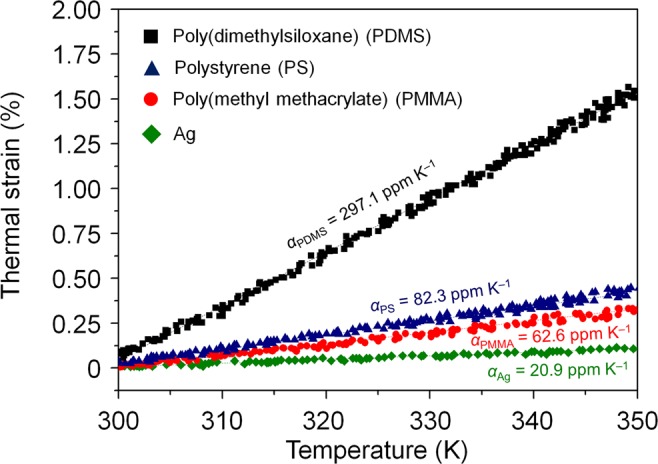
Thermal expansion behavior of polymer and metal thin films. For polymer films, the thin films of PDMS, PS and PMMA are tested. The thermal expansion of Ag thin films is explored for metal thin films. The thickness of PDMS, PS, PMMA and Ag thin films are 5000, 80, 200, and 500 nm, respectively. The CTE is obtained by a linear fitting of a thermal strain-temperature curve.

The in-plane thermal expansion of various polymer and metal thin films including PDMS, PS, PMMA, and Ag, which are hydrophobic and non-wetting materials, was examined without any solid substrate. The thermal strain-temperature curve of each film shows good linearity during water heating (Fig. 2). The CTEs were determined by linear fitting and calculating of the slope of the curves. The CTEs of PDMS, PS, PMMA, and Ag were 297.1 ± 8.8, 82.3 ± 1.6, 62.6 ± 1.4, and 20.9 ± 0.8 ppm K^−1^, respectively. Each CTE value represents the average of five specimens tested. The good agreement with reference values (Supplementary Table S1)^16–23^ verifies the accuracy and precision of our measurement method. Again, the in-plane CTE measurements on the water surface did not require extra calculations and physical properties such as the dielectric function of thin films. In conventional CTE measurements such as spectroscopic ellipsometry, X-ray reflectometry, and neutron reflectometry, iterative calculations that can minimize errors are needed because the physical properties are often affected by the temperature or film thickness^13,14^. Our CTE measurement method does not require variables that depend on the temperature or the film thickness, enabling direct, rapid and precise CTE measurement of the thin films.

### In-plane thermal expansion of PS thin films

**Figure 3:**
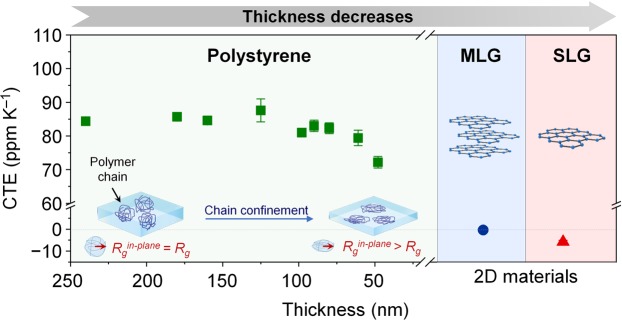
Coefficient of thermal expansion of PS thin films and graphene. (PS, green) CTE of PS thin films with the thickness ranged from 48 nm to 240 nm. When the thickness of PS thin film become comparable to the sixth of the radius of gyration for polymer chains 6*R*_*g*_ (about 90 nm), the polymer chains of the PS films are deformed in parallel to the film surface. Then the radius of gyration for in-plane direction *R*_*g*_^*in-plane*^ becomes larger than *R*_*g*_ (*R*_*g*_^*in-plane*^ > *R*_*g*_). When the polymer chains are deformed, the chains become hardened due to the stress. The stress-induced hardening causes the CTE reduction of the PS thin films. (Graphene, blue and red) The CTEs of multi-layer graphene (MLG) and single-layer graphene (SLG) show negative values, −0.4 ± 0.2 and −5.8 ± 0.7 ppm K^−1^, respectively. The MLG shows higher CTE than the SLG, because the thermal contraction is restricted by the stacked graphene layers.

Next, the in-plane CTE measurement method was applied to tens of nanometer thick PS films and even multi and single-layer graphene (Fig. 3). PS films with various thicknesses, as well as graphene, can expand freely on the water surface due to the lack of substrate constraint. The CTE of PS films remains nearly constant above a thickness of 90 nm, however below 90 nm, it starts to decrease as the film thickness decreases. The thickness dependence of CTE for PS thin films can be explained by the chain confinement effect^1,2,5,7,8,24^. When the thickness of the PS film becomes comparable to the radius of gyration *R*_*g*_, which characterizes the dimensions of a polymer chain, the chains become deformed^25–27^ and hardened due to the stress^2,24^. According to Inoue *et al*., stress-induced hardening causes the reduction of CTE as the film thickness decreases^2^. Thermal expansion originates from the anharmonic potential energy of the material. The anharmonicity causes an increase in the interatomic separation as the temperature increases^28^. Inoue *et al*.^2^ suggest a relationship between the CTE *α* and harmonic force constant *f*, *α ~ f*^−2^. The force constant *f* increases when stress-induced hardening occurs because of the confinement effect, therefore, CTE *α* becomes smaller^2,24^.

In previous studies, the critical thickness where the chain confinement effect begins to appear has been reported as 2*R*_*g*_^2,8,24^. However, according to our results, the thickness where the CTE of PS thin films started to decrease was larger than 2*R*_*g*_, which was approximately 30 nm, assuming that the end-to-end distance of the polymer chain was 2*R*_*g*_^29^. The difference in the critical thickness can result from the difference in the direction of the measured thermal strain. In our study, the in-plane length was measured as the thermal strain, while the thickness change was measured in previous studies. In the thickness direction, the polymer chains started to fold towards the center^26^ when the film thickness was smaller than 2*R*_*g*_. On the other hand, in the in-plane direction, chain elongation parallel to film surface occurs and the in-plane component of the radius of gyration *R*_*g*_^*in-plane*^ becomes larger than the radius of gyration *R*_*g*_ (Fig. 3) when the film thickness is smaller than 6*R*_g_^25^, that is about 90 nm in our study. Therefore, reduction of CTE was observed near 6*R*_*g*_ because our method measured in-plane thermal strain and stress-induced hardening of the polymer chain in the in-plane direction occurred below thickness of 6*R*_*g*_.

### CTE measurement of graphene without substrate

**Figure 4:**
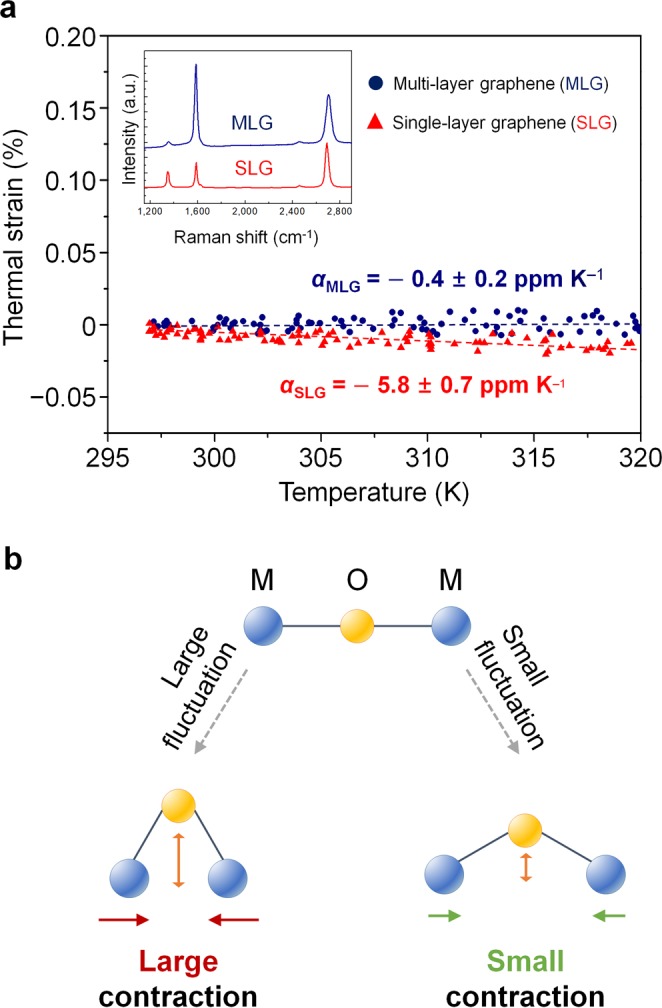
Negative thermal expansion of multi and single-layer graphene. (**a**) Thermal strain-temperature curves of SLG and MLG during the heating. The CTEs of SLG and MLG are obtained from the slope of the curves. The SLG contracts more and shows lower CTE than the MLG. The inset graph shows Raman spectroscopy in SLG and MLG. The peak intensity ratio of 2D peak (~ 2700 cm^−1^) to G peak (~ 1580 cm^−1^) characterizes the number of graphene layers. (**b**) Schematic illustration of the M-O-M bond to explain negative thermal expansion due to the out-of-plane thermal fluctuation. M and O usually represent metal and oxygen atoms, respectively. The transverse vibration of the O atom induces the decrease in the distance between the two M atoms. Due to the decrease in the M-M distance, the material shows the thermal contraction during the heating. The larger the fluctuation of the O atom, the larger the thermal contraction. The transverse vibration of the MLG is restricted by the adjacent graphene layers, therefore, the thermal contraction of the MLG is less than that of the SLG.

Again, our CTE measurement method can explore the in-plane thermal behavior of thin films without substrate constraint. The in-plane CTE measurement method becomes more powerful when the material becomes extremely thin. The CTE of chemical-vapor-deposited single-layer graphene (SLG) and multi-layer graphene (MLG) were examined (Fig. 4a). The number of layers of graphene specimen was identified by the peak intensity ratio of the 2D peak (~2700 cm^−1^) to the G peak (~1580 cm^−1^), obtained from Raman spectroscopy (Fig. 4a, inset)^30^. Both the SLG and MLG showed negative CTE values, which were −5.8 ± 0.7 ppm K^−1^ for SLG and −0.4 ± 0.2 ppm K^−1^ for MLG in the temperature range of 297 to 320 K (Fig. 4a). A negative CTE means the graphene shows in-plane contraction during heating. Thermal contraction originates from the transverse vibration of carbon atoms^3,31,32^. In-plane contraction due to the transverse vibration can be simply explained with the M-O-M bond model (Fig. 4b). The transverse vibration of the bridging O atom results in a decrease of distance between the two M atoms. If the fluctuation of transverse vibration is reduced, the decrease in the M-M distance will also be reduced^31^. Similarly, in graphene, the transverse vibration of carbon atoms leads to a decrease in the in-plane lattice parameter, and eventually, thermal contraction occurs. The transverse vibration of multi-layer graphene may be restricted due to constraints from adjacent graphene layers^32–35^. Therefore, the thermal contraction of MLG was less than that of SLG_,_ and the CTE of MLG showed a value smaller than that of SLG from 297 to 320 K^3,4,32^.

In summary, we developed a method of in-plane CTE measurement for thin films, and even a one-atom-thick film on a water surface, by using the DIC technique. The water surface provided frictionless sliding during the thermal expansion of the thin film. Therefore, in-plane CTE measurement was enabled without any substrate constraints. The in-plane thermal strain was measured directly by using the DIC technique to calculate length changes of the thin film. For PS thin films, the thickness dependence of the in-plane CTE was observed due to hardening from in-plane polymer chain deformation. Moreover, thermal contraction of the multi and single-layer graphene was observed directly without the constraints. The in-plane CTE measurement method can elucidate the in-plane thermal behavior of thin films, whereas the conventional method usually delivers out-of-plane CTE for supported films. We believe our method, by minimizing the substrate effect, can improve understanding of the intrinsic thermal behavior of ultra-thin films such as two-dimensional materials or atomic layer deposition films.

## Methods

### Substrate preparation

500 nm of Cu sacrificial layer was evaporated on a thermally oxidized silicon wafer. 300 nm of silicon dioxide (SiO_2_) was formed by wet thermal oxidation. The wafer was cleaved into 15 mm × 15 mm samples. For substrate cleaning, O_2_ plasma treatment was carried out for 1 min with 100 W of power.

### Fabrication of PDMS films

The curing agent and the base were mixed with a weight ratio of 1:10 (Sylgard 184, Dow Corning, USA). After a degassing process in a vacuum environment, the mixed solution was spin-coated onto Cu/SiO_2_/Si substrate at 5000 rpm for 1 min. Then the PDMS thin film on the substrate was annealed at 353 K for 2 hours using a convection oven.

### Fabrication of PMMA films

PMMA (950 PMMA A4, MicroChem, USA) was spun onto Cu/SiO_2_/Si substrate at 5,000 rpm for 1 min. The PMMA on the substrate was annealed at 453 K for 3 min in the vacuum oven.

### Fabrication of PS films

PS (molecular weight 192 k, Sigma-Aldrich, USA) was dissolved into toluene by stirring. The solution was spin-coated onto Cu/SiO_2_/Si substrate for 1 min. The spin speed was varied from 500 to 5000 rpm to control the thickness of the PS thin films. Then the PS film on the substrate was annealed at 433 K for 8 hours using the vacuum oven. Before peeling, each specimen was cut to a size of 10 mm × 13 mm by removing the edge of the specimen.

## Supplementary information

Supplementary Information

## Data Availability

The datasets generated during and/or analysed during the current study are available from the corresponding author on reasonable request.
