# Supplementary material for: Thermal expansion behavior of thin films expanding freely on water surface
Source: Sci Rep. 2019 May 8;9:7071. doi: 10.1038/s41598-019-43592-x (PMC6506477; doi:10.1038/s41598-019-43592-x)
Supplement: Supplementary file 1 — Supplementary Information [file 41598_2019_43592_MOESM1_ESM.pdf]

Supplementary Information

Thermal expansion behavior of thin films expanding freely on water surface

Jae-Han Kim[1,2†], Kyung-Lim Jang[1†], Kwangho Ahn[1], Taeshik Yoon[1], Tae-Ik Lee[1], and Taek-Soo Kim[1*]

[1]Department of Mechanical Engineering, KAIST, Daejeon, 34141, Korea

[2]Korea Atomic Energy Research Institute, Daejeon, 34057, Korea

[*]E-mail: tskim1@kaist.ac.kr

Contents

Fig. S1: Preparation of thin film specimen floating on water surface.

Fig. S2: Heating simulations for thin films.

Fig. S3: Measurement of water temperature during heating at different positions.

Table S1: Coefficient of thermal expansion of polymer and metal thin films.

Supplementary Figure S1

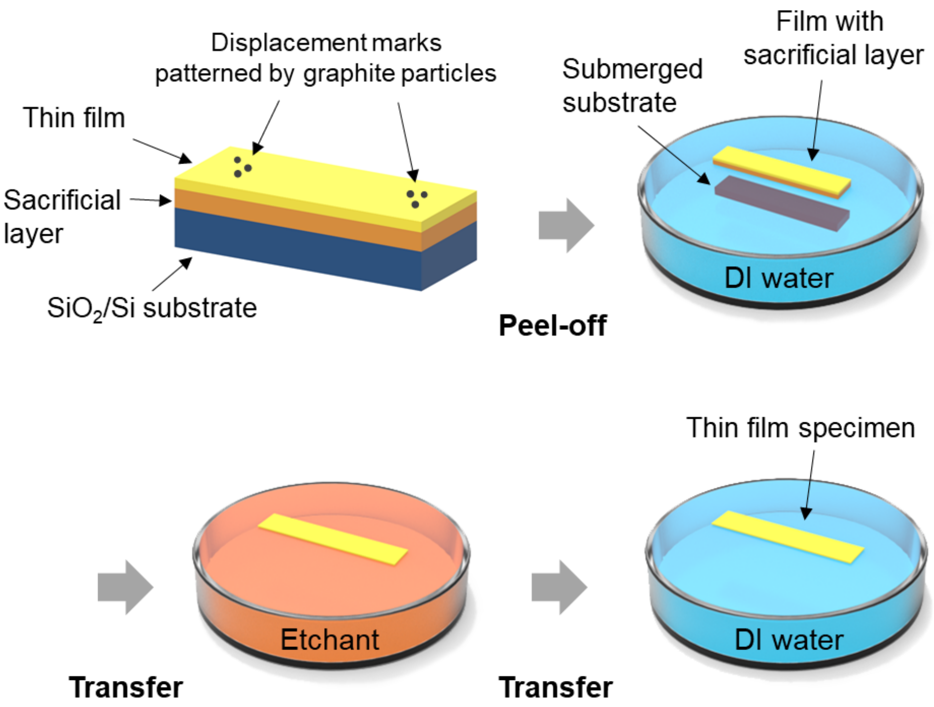

Fig. S1. Preparation of thin film specimen floating on water surface. Specimen preparation procedures for floating thin film on deionized (DI) water. Thin film is fabricated on a Cu/SiO_2/Si substrate. Fine graphite particles are deposited on the thin film as displacement marks. The patterned thin film specimen on the Cu sacrificial layer is peeled off with the assistance of the water surface. Then the thin film on the Cu layer is transferred to Cu etchant. The Cu layer is etched by the Cu etchant, a 0.05 M ammonium persulfate solution. After the etching is completed, the thin film specimen is transferred on the DI water by the scoop-up method.

Supplementary Figure S2

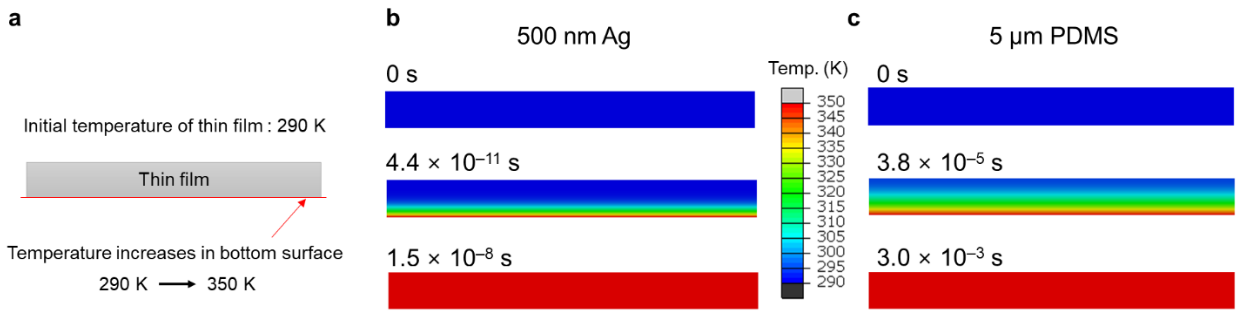

Fig. S2: Heating simulations for thin films. (**a**) Temperature change is simulated by heating the bottom surface of the thin film up to 350 K using Abaqus/CAE 6.14 software. The initial temperature of the thin film is 290 K. Then the temperature of the bottom surface is changed to 350 K instantaneously. It is assumed that the thin film is in contact with the water surface at the bottom. (**b**,**c**) The simulation results of 500 nm Ag and 5 μm PDMS. (**b**) The temperature of Ag film reaches 350 K after 1.5×10^{-8} s. (**c**) The PDMS film is fully heated after 3.0×10^{-3} s. In both cases, the thin films are fully heated up to 350 K immediately. Therefore, the temperature of the thin film can be considered same as the temperature of the water surface. Furthermore, the temperature deviation in out-of-plane direction can be negligible.

Supplementary Figure S3

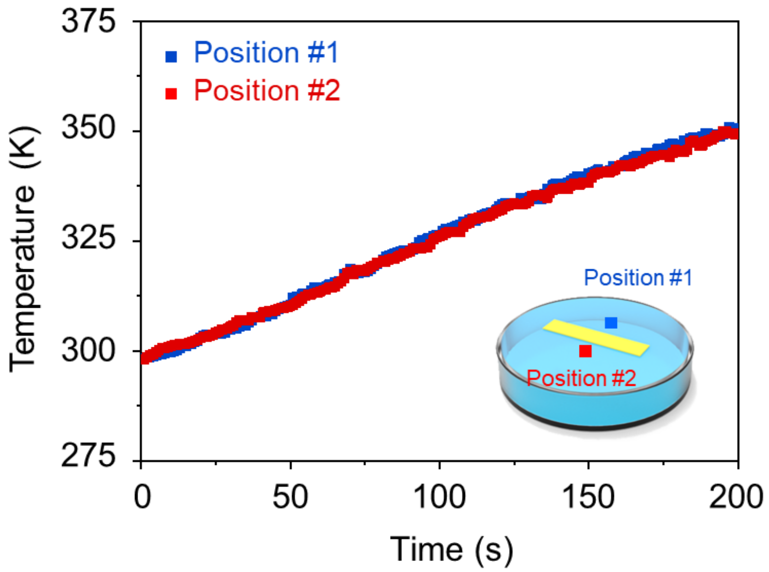

Fig. S3: Measurement of water temperature during heating at different positions. Temperature measurement conducted at two different positions of the center of the dish shows almost the same increase during heating. Therefore, the temperature deviation at different positions is almost negligible.

Supplementary Table S1: Coefficient of thermal expansion of polymer and metal thin films.

Materials	Thickness	CTE (ppm K^{-1})	
		Measured	Reference
PDMS	5 μm	297.1 ± 8.8	266.5 – 310[16,17]
Polystyrene	80 nm	82.3 ± 1.6	60 – 80[18]
PMMA	200 nm	62.6 ± 1.4	50 – 70[19-22]
Ag	500 nm	20.9 ± 0.8	19[23]
